# Association of ultra-early diffusion-weighted magnetic resonance imaging with neurological outcomes after out-of-hospital cardiac arrest

**DOI:** 10.1186/s13054-023-04305-z

**Published:** 2023-01-13

**Authors:** Changshin Kang, Jin Hong Min, Jung Soo Park, Yeonho You, Wonjoon Jeong, Hong Joon Ahn, Yong Nam In, In Ho Lee, Hye Seon Jeong, Byung Kook Lee, Jinwoo Jeong

**Affiliations:** 1 Department of Emergency Medicine, Chungnam National University Hospital, Daejeon, Republic of Korea; 2 Department of Emergency Medicine, College of Medicine, Chungnam National University, 266 Munwha-ro, Jung-gu, Daejeon, 35015 Republic of Korea; 3 Department of Radiology, College of Medicine, Chungnam National University, 266, Munhwa-ro, Jung-gu, Daejeon, Republic of Korea; 4 Department of Neurology, Chungnam National University Hospital, Daejeon, Republic of Korea; 5 Department of Emergency Medicine, Chonnam National University Medical School, Gwangju, Republic of Korea; 6 Department of Emergency Medicine, Dong-A University College of Medicine, Busan, Korea

**Keywords:** Out-of-hospital cardiac arrest, Diffusion magnetic resonance imaging, Cerebrospinal fluid, Prognosis

## Abstract

**Background:**

This study aimed to investigate the association between ultra-early (within 6 h after return of spontaneous circulation [ROSC]) brain diffusion-weighted magnetic resonance imaging (DW-MRI) and neurological outcomes in comatose survivors after out-of-hospital cardiac arrest.

**Methods:**

We conducted a registry-based observational study from May 2018 to February 2022 at a Chungnam national university hospital in Daejeon, Korea. Presence of high-signal intensity (HSI) (P_HSI_) was defined as a HSI on DW-MRI with corresponding hypoattenuation on the apparent diffusion coefficient map irrespective of volume after hypoxic ischemic brain injury; absence of HSI was defined as A_HSI_. The primary outcome was the dichotomized cerebral performance category (CPC) at 6 months, defined as good (CPC 1–2) or poor (CPC 3–5).

**Results:**

Of the 110 patients (30 women [27.3%]; median (interquartile range [IQR]) age, 58 [38–69] years), 48 (43.6%) had a good neurological outcome, time from ROSC to MRI scan was 2.8 h (IQR 2.0–4.0 h), and the P_HSI_ on DW-MRI was observed in 46 (41.8%) patients. No patients in the P_HSI_ group had a good neurological outcome compared with 48 (75%) patients in the A_HSI_ group. In the A_HSI_ group, cerebrospinal fluid (CSF) neuron-specific enolase (NSE) levels were significantly lower in the group with good neurological outcome compared to the group with poor neurological outcome (20.1 [14.4–30.7] ng/mL vs. 84.3 [32.4–167.0] ng/mL, *P* < 0.001). The area under the curve for P_HSI_ on DW-MRI was 0.87 (95% confidence interval [CI] 0.80–0.93), and the specificity and sensitivity for predicting a poor neurological outcome were 100% (95% CI 91.2%–100%) and 74.2% (95% CI 62.0–83.5%), respectively. A higher sensitivity was observed when CSF NSE levels were combined (88.7% [95% CI 77.1–95.1%]; 100% specificity).

**Conclusions:**

In this cohort study, P_HSI_ findings on ultra-early DW-MRI were associated with poor neurological outcomes 6 months following the cardiac arrest. The combined CSF NSE levels showed higher sensitivity at 100% specificity than on DW-MRI alone. Prospective multicenter studies are required to confirm these results.

**Supplementary Information:**

The online version contains supplementary material available at 10.1186/s13054-023-04305-z.

## Background

In the United States, > 356,000 cases of out-of-hospital cardiac arrest (OHCA) occur annually, of which nearly 90% are fatal [1]. Most deaths result from withdrawal of life-sustaining treatment (WLST) based on a predicted poor neurological outcome. Approximately 19% of patients post-cardiac arrest (CA) die due to early WLST (within three days post-CA), despite a predicted good neurological outcome [2–4]. International guidelines for post-CA care recommend that neurological prognostication should be delayed at least 72 h after return of spontaneous circulation (ROSC) [5, 6]. Nevertheless, WLST is occasionally performed earlier than 72 h after ROSC due to medical factors, patient values and preferences or premature neurological prognostication related to intensive care unit (ICU) admission and for patients with severe hypoxic ischemic brain injury (HIBI) [3–5, 7]. Furthermore, ICU overcrowding due to the recent coronavirus disease pandemic (COVID-19) has impeded the provision of adequate treatment opportunities for patients with a potentially good neurological prognosis [8]. Therefore, early and accurate prediction of the neurological prognosis in CA survivors is important to enable medical resources to be appropriately distributed and to prevent hasty WLST in patients with neurological recovery potential [9]. However, no reliable tools are available to help clinicians predict neurological outcomes early [10–14].

Several studies have aimed to predict neurological outcome during the early stage (i.e., before targeted temperature management [TTM]) using neuroimaging examinations, such as computed tomography (CT) and magnetic resonance imaging (MRI) [7, 15–18]. Some studies have reported limitations in the use of the gray-white matter ratio (GWR) on brain CT as an early prognostic tool [15, 16]. In contrast, high-signal intensity (HSI) (“restricted diffusion”) in ultra-early (within 6 h after ROSC) diffusion-weighted MRI (DW-MRI) has been reported as a useful tool for predicting neurological outcome early stage [9, 17, 18]. Neuron-specific enolase (NSE), a biomarker obtained from cerebrospinal fluid (CSF), has also been reported to be a useful prognostic tool in the early stage [19]. However, these studies were limited by their small sample sizes, and a combination approaches using other tools were rare investigated.

This study aimed to assess the prognostic value of ultra-early DW-MRI in a non-WLST setting assessed by the presence or absence of HSI. We also evaluated the combinations that can improve predictive power for poor neurological outcome six months after cardiac arrest.

## Methods

### Study design and patients

This retrospective observational study used prospectively collected data from adult (aged ≥ 18 years) comatose OHCA survivors treated with TTM at a single tertiary hospital from May 2018 to January 2022. This study was approved by our Institutional Review Board (CNUH–2022–05–013), and written informed consent was obtained from all patients and/or their legal guardian(s) in accordance with national requirements and the principles of the Declaration of Helsinki, and registered in a database.

Inclusion criteria comprised adult OHCA survivors who received DW-MRI within 6 h of ROSC prior to TTM. Exclusion criteria comprised patients: (i) whose MRI scanning time exceeded 6 h after ROSC, (ii) who had experienced a traumatic CA, (iii) who had received extracorporeal membrane oxygenation, and (iv) whose cause of the presence of HSI (P_HSI_) on DW-MRI was not due to HIBI (e.g., cerebral infarction).

### Post-cardiac arrest care

All the included patients for this study underwent post-cardiac arrest care bundle including TTM. They who were unable to obey commands (Glasgow Coma Scale motor score of less than 6) with a target temperature of 33 or 36 °C, except those with active bleeding, refractory hemodynamic instability, possible causes of coma other than cardiac arrest, terminal malignancy, or poor pre-arrest neurologic status (Cerebral Performance Category [CPC] 3 or 4). TTM was performed using cooling devices (Arctic Sun® 5000, BD, Franklin Lakes, NJ, USA). The targeted temperature of 33 or 36 °C was maintained for 24 h with rewarming to 37 °C at the rate of 0.25 °C per an hour and it was monitored using an esophageal or bladder temperature probe. Target temperature was determined by the attending physician (33 vs. 36 °C) according to hemodynamic status or cardiac arrest characteristics. If there was evidence of electrographic seizure or a clinical diagnosis of seizure, anti-epileptic drugs were administered; benzodiazepine and/or levetiracetam. All patients received standard intensive care according to our institutional intensive care unit protocol based on the 2021 international guidelines for post-cardiac arrest care. WLST was not permitted prior to February 2018 in South Korea unless a patient had been pronounced brain-dead, and WLST has since been performed rarely. In this study, WLST during TTM did not occur, although some patients were pronounced dead according to circulatory or neurological criteria despite maximal support.

### Data collection

We extracted the following data from our prospective registry**:** age, sex, Charlson comorbidity index (CCI), witnessed collapse, bystander cardiopulmonary resuscitation (CPR), time from CPR to the ROSC (low flow time), first monitored rhythm, etiology of cardiac arrest, time to obtain biomarkers (NSE and/or albumin), time to perform CT and MRI from ROSC, the potential indicator for systemic injury severity (pH and lactic acid immediately after ROSC), targeted temperature for TTM, and seizure like movement observed before use of anti-epileptic drug or sedative agents. In addition, we extracted data of predictors measured within 6 h from ROSC: serum and CSF NSE levels, albumin quotient (albumin_[CSF]/_albumin_[serum]_) (Q_A_), the percentage of voxels (PV) below 650 × 10^−6^ mm^2^/s apparent diffusion coefficient (ADC) thresholds per total voxel (PV 650), and GWR on brain CT obtained within 6 h after ROSC.

**Fig. 1:**
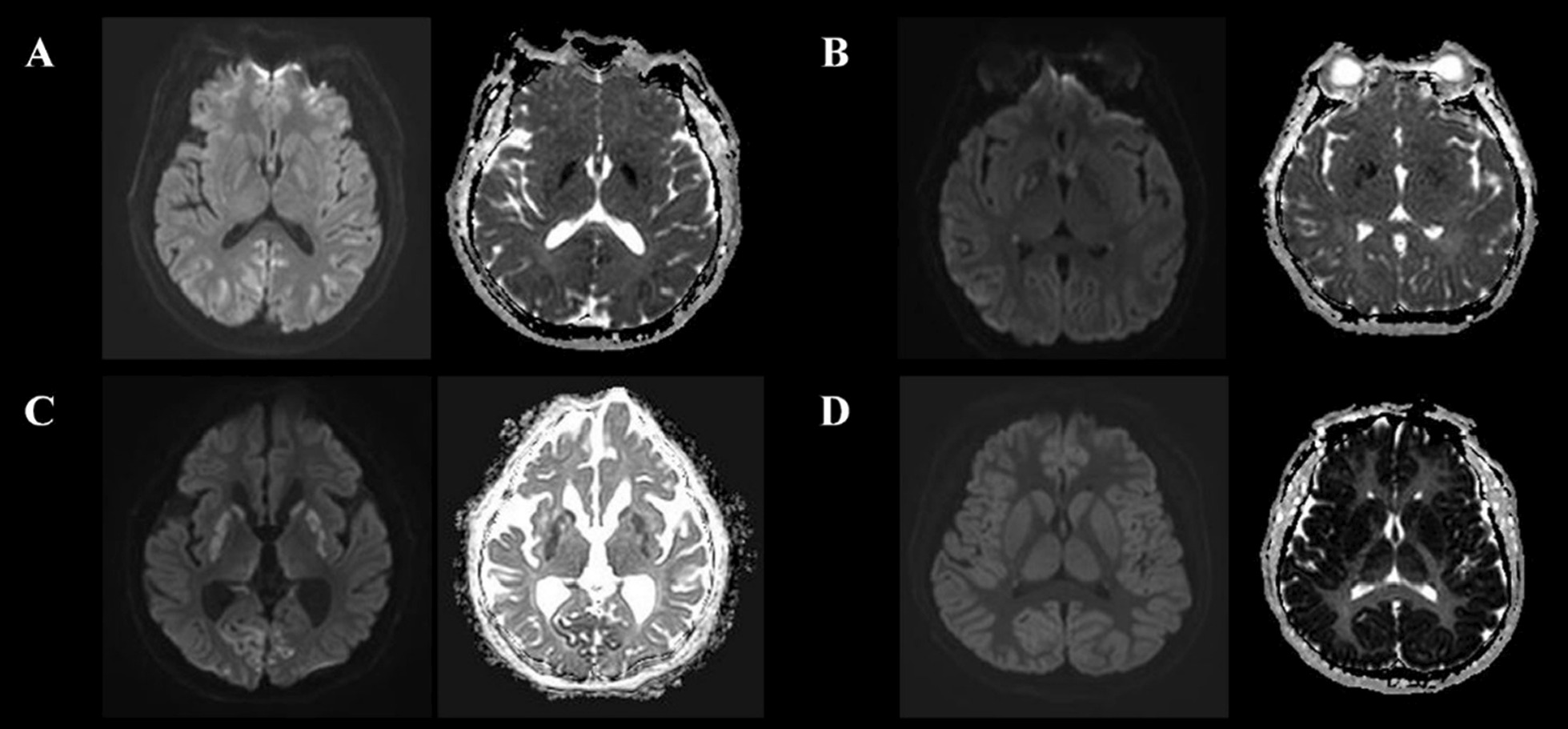
Various pattern of ultra-early DW-MRI and ADC of OHCA survivals. **A**. A 70-years old male. Gyriform restrictive diffusion in occipital cortex. **B**. A 31-years old female. Gyriform and regional restrictive diffusion in occipital cortex, temporal cortex, and deep gray matter, respectively. **C**. A 74-years old male. Gyriform and regional restrictive diffusion in occipital cortex and deep gray matter, respectively. **D**. A 58-years old male. Extensive gyriform resticted diffusion in all gray matter. *Abbreviations:* *OHCA*, out-of-hospital cardiac arrest; *DW-MRI*, diffusion-weighted magnetic resonance imaging; *ADC*, Apparent diffusion coefficient

MRI scanning was performed using a 3 T scanner (Achieva, Philips Healthcare, Amsterdam, The Netherlands) and included DW-MRI, ADC measurements, and T2-weighted imaging. Forty consecutive DW-MRI sections per patient were acquired using standard *b* = 1000 s/mm^2^, and it was independently assessed by a neuroradiologist (I.H.L) and neurologist (H.S.J.) completely blinded to clinical information. P_HSI_ was defined as a HSI on DW-MRI with corresponding hypoattenuation on the ADC map irrespective of volume after HIBI, and other cases as absence of HSI (A_HSI_) (Fig. 1) [20–22], and when there was a difference of opinion, it was resolved by consensus (see Additional file 1). However, patients with single or multiple HSI confined to a specific vascular territory on DW-MRI were excluded from this study because they were not presumed HIBI [23]. The % voxels of ADC threshold is defined as the PV below a different ADC threshold per total voxels, PV 650 was calculated as the percentage brain volume with voxels below 650 (< 650 × 10^−6^ mm^2^/s) ADC value. In addition, average ADC value is defined as the mean ADC of the entire brain. Lumbar catheter placement was performed using a Hermetic™ Lumbar Catheter Accessory Kit (Integra Neurosciences, Plainsboro, NJ, USA), and CSF samples and ICP were measured using a LiquoGuard® pump system (Möller-Medical, Fulda, Germany). To measure the NSE level, an electro-chemi-luminescence immunoassay kit (COBAS® e801, Roche Diagnostics, Rotkreuz, Switzerland) was used. To determine the extent of blood–brain barrier (BBB) disruption, we used an albumin quotient (Q_A_) = albumin_[CSF]_/albumin_[serum]_ value [24]. Brain non-contrast CT scans were obtained in 5-mm slices using a 64-channel system (Somatom Sensation 64, Siemens Healthineers, Munich, Germany), and a neuroradiologist (I.H.L) measured the Hounsfield units (HU) of the putamen (P), caudate nucleus (CN), posterior limb of the internal capsule (PIC), and corpus callosum (CC) to calculate the GWR ([P + CN]/[PIC + CC]).

### Outcome

Neurological outcome was assessed at 6 months later from OHCA using the CPC score. The CPC score classifies patients into 5 categories: CPC 1 (good performance), CPC 2 (moderate disability), CPC 3 (severe disability), CPC 4 (vegetative state), or CPC 5 (brain death or death). It was performed either through face-to-face interviews or telephone interviews, which shown almost perfect agreements in inter- and intra-rater reliabilities [25]. The primary outcome was a poor neurological outcome, defined as from CPC 3 to 5.

### Statistical analysis

Categorical variables were described as frequencies with percentiles. Continuous variables were described as median values with interquartile ranges (IQRs) as all continuous variables had a non-normal distribution. We compared categorical variables between the groups using *χ*^2^ tests with continuity correction in 2 × 2 tables or a Fisher’s exact test, as appropriate. We compared continuous variables between two groups using a Mann–Whitney U test. Receiver operating characteristic (ROC) analyses were performed to assess the prognostic performances of single predictors and their combination models. Comparison of the area under the ROC curves (AUC) were performed using the DeLong test [26]. Combination models were constructed using logistic regression analysis. Sensitivity, specificity, positive predictive value (PPV) and negative predictive value (NPV) for poor neurological outcomes at 6 months were calculated using the Agresti-Coull 95% confidence intervals (CIs) [27]. The optimal cut-off values for predicting poor neurological outcomes were determined using 100% specificity. The AUC values of 0.50–0.69, 0.70–0.79, 0.80–0.89, and 0.90–1.00 indicated poor, fair, good, and excellent prognostic performance, respectively [28]. Inter-rater reliability was determined using Cohen’s kappa (k) for nominal variables, such as the presence/absence of HSI on DW-MRI. The kappa values of 0.01–0.2, 0.21–0.40, 0.41–0.60, 0.61–0.80, and 0.81–1.00 indicated slight, fair, moderate, substantial, and almost perfect agreement, respectively [29]. Data were analyzed using IBM SPSS Statistics 26.0 for Windows (IBM Corp., Armonk, NY). The AUC were calculated using MedCalc version 15.2.2 (MedCalc Software, Mariakerke, Belgium). The Agresti-Coull CIs were calculated using R 4.1.0 (R Foundation for Statistical Computing, Vienna, Austria, 2021) and the package “binom” (Sundar Dorai-Raj, 2022), *P*-values < 0.05 were considered statistically significant at 95% CIs.

## Results

### Baseline characteristics of participants

**Fig. 2:**
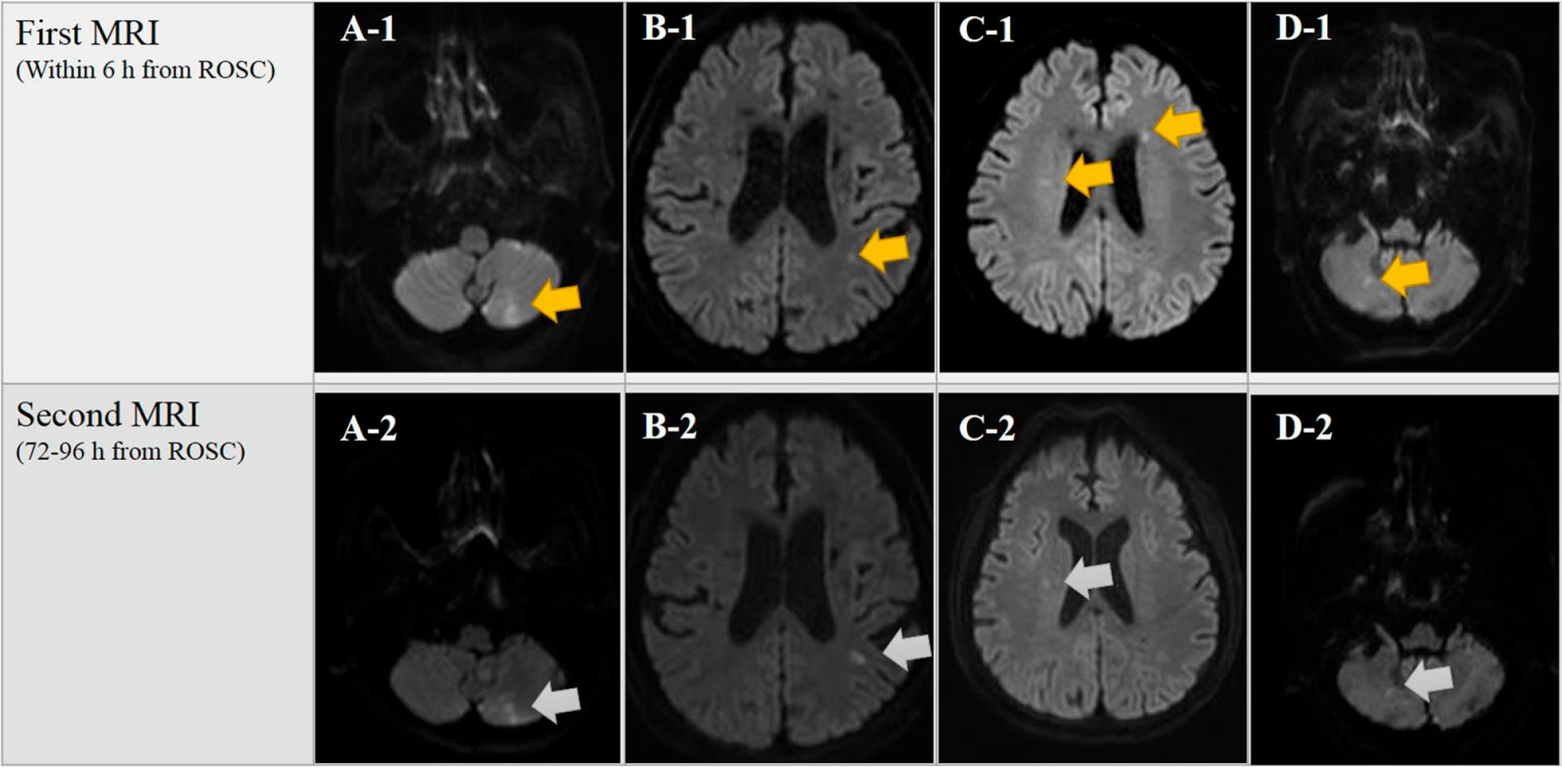
Patients excluded from this study despite DW-MRI showing the presence of HSI. Our institution's targeted temperature management protocol recommends but does not require obtaining two brain MRI scans within 6 h (first MRI) and between 72 and 96 h (second MRI) after ROSC. In this study, 4 patients showed only 1 or 2 focal HSI (orange arrow) on the ultra-early DW-MRI, and all of them did not exhibit an expanded HSI (gray arrow) area in the DW-MRI 3–4 days after ROSC. They all showed good neurological outcome, and among them, cases A, B, and D showed higher CPC scores (CPC 1) compare with case C (CPC 2). *Abbreviations:* *CPC*, cerebral performance category; *DW-MRI*, diffusion-weighted magnetic resonance imaging; *HSI*, high-signal intensity; *MRI*, magnetic resonance imaging; *ROSC*, return of spontaneous circulation

**Fig. 3:**
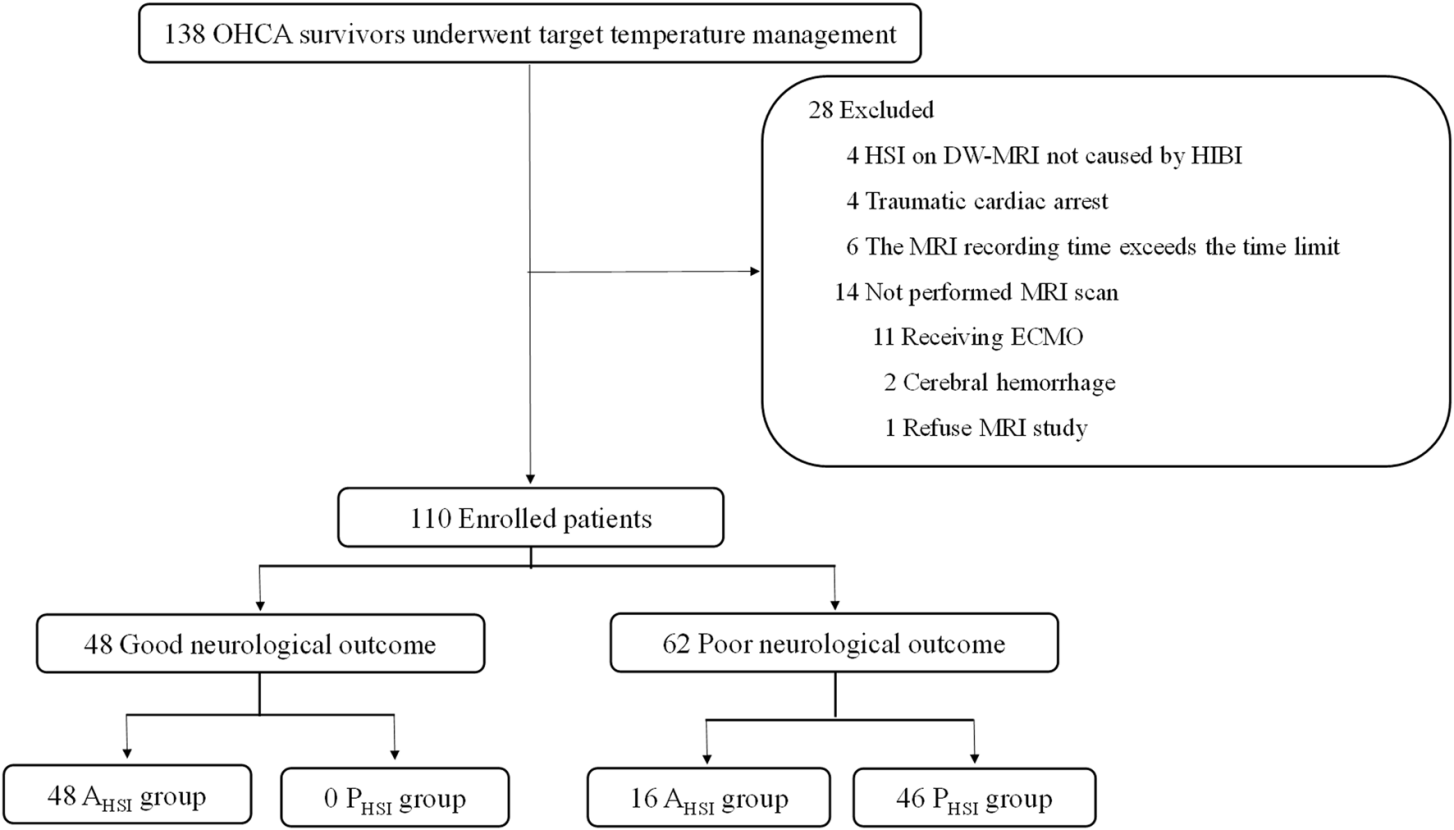
Flow diagram of the included study patients. P_HSI_, presence of HSI on DW-MRI; A_HSI_, absence of HSI on DW-MRI. *Abbreviations:* *DW-MRI*, diffusion-weighted magnetic resonance imaging; *ECMO*, extracorporeal membrane oxygenation; *HIBI*, hypoxic ischemic brain injury; *HSI*, high-signal intensity; *MRI*, magnetic resonance imaging

In total, 138 OHCA survivors who had undergone TTM were recorded during the study period. Of these, P_HSI_ in four patients was not due to HIBI (Fig. 2), four patients had a CA due to trauma, six patients had an MRI scan 6 h after ROSC, and MRI scans had not been performed in 14 patients. Thus, 110 patients were included. Six months after ROSC, 48 (44%) and 62 (56%) patients were assigned to good and poor neurological outcome groups, respectively (Fig. 3). The inter-rater reliability for the presence/absence of HSI in DW-MRI showed almost perfect agreement (*k* = 0.87; see Additional file 1, Table S1). No patients in the P_HSI_ group had a good neurological outcome compared with 48 (75%) patients in the A_HSI_ group.﻿ In the P_HSI_ group, a multi-regional involvement of HSI was most observed at 43.5%, followed by 22% with the global involvement and 8.7% with the regional involvement and multi-focal pattern, respectively; and HSI was most observed in an occipital lobe at 26.0%, followed by 22.5% in temporal lobe, 22.0% in deep gray matter, 17.3% in parietal lobe, and 12.1% in frontal lobe (Table 1 and see Additional file 1: Figure S1). Demographic and OHCA characteristics stratified according to neurological outcome are shown in Table 2. No differences were found between the groups with good and poor neurological outcomes in terms of age, sex, CCI, and time to obtain biomarker samples, CT, and MRI performed after ROSC. No adverse events or complications were found to be associated with ultra-early MRI scanning during the study period.

**Table 1:** Classification of hypoxic ischemic brain injury according to the lesion visualized on diffusion-weighted magnetic resonance imaging and corresponding apparent diffusion coefficient map and number of cases according to the anatomical location of restricted diffusion

Classification	Numbers (%)	Location of restricted diffusion				
		Frontal lobe	Temporal lobe	Parietal lobe	Occipital lobe	Deep grey matter
Regional involvement	4 (8.7)	0	0	0	4	0
Multi-regional involvement	20 (43.5)	3	17	8	19	16
Multi-focal pattern	4 (8.7)	0	4	4	4	4
Global involvement	18 (39.1)	18	18	18	18	18
Total	46 (100.0)	21	39	30	45	38

**Table 2:** Baseline demographic data and arrest characteristics

Characteristic	Patients, no. (%)				Patients, no. (%)		
	Overall cohort (*n* = 110)	Good neurological outcome (*n* = 48)	Poor neurological outcome (*n* = 62)	*P*-value^b^	A_HSI_ group (*n* = 64)^a^	P_HSI_ group (*n* = 46)^a^	*P*-value^b^
Age, median (IQR), *y*	57.5 (38.0–69.0)	58.5 (38.0–68.0)	57.5 (41.8–69.0)	.75	57.5 (30.5–67.5)	56.5 (48.3–72.3)	.25
*Sex*							
Female	30 (27.3)	10 (20.8)	20 (32.3)	.20	16 (25.0)	14 (30.4)	.67
Male	80 (72.7)	38 (79.2)	42 (67.7)		48 (75.0)	32 (69.6)	
CCI score, median (IQR)	2 (0–4)	2 (0–4)	2 (0–4)	.69	2 (0–4)	2 (1–4)	.34
*Arrest characteristics*							
Witness	61 (55.5)	39 (81.3)	22 (35.5)	< 0.001	44 (68.8)	17 (37.0)	< 0.001
Bystander CPR	77 (70.0)	39 (81.3)	38 (61.3)	.03	49 (76.6)	28 (60.9)	.09
Shockable rhythm	36 (32.7)	30 (62.5)	6 (9.7)	< 0.001	33 (51.6)	3 (6.5)	< 0.001
Cardiac etiology	42 (38.2)	30 (62.5)	12 (19.4)	< 0.001	32 (50.0)	10 (21.7)	.003
Low flow time, median (IQR), min	20.0 (9.5–30.0)	12.5 (8.0–18.8)	29.5 (20.5–43.0)	< 0.001	15.0 (9.0–18.8)	27.5 (20.0–39.8)	< 0.001
*Post-cardiac arrest care*							
Target temperature, 33 °C	98 (89.1)	40 (83.3)	58 (93.5)	.12	56 (87.5)	42 (91.3)	.76
Early PCI, *n* (%)	9 (8.2)	6 (12.5)	3 (4.8)	.18	9 (14.1)	0	.01
Seizure before MRI, *n* (%)	28 (25.5)	12 (25.0)	16 (25.8)	.96	20 (31.3)	8 (17.4)	.12
*Laboratory results after ROSC, median (IQR)*							
pH	7.20 (7.06–7.30)	7.25 (7.13–7.34)	7.14 (7.00–7.27)	.004	7.25 (7.13–7.33)	7.12 (6.99–7.23)	< 0.001
Lactic acid, mmol/L	8.2 (4.5–11.0)	7.0 (3.6–10.4)	8.7 (4.2–11.0)	.07	7.0 (4.0–7.0)	9.8 (6.4–11.0)	.03
*Times to examinations, median (IQR), h*							
ROSC to CT, 95^c^	1.3 (0.7–2.2)	1.1 (0.6–1.8)	1.5 (0.9–2.4)	.17	1.1 (0.5–1.7)	1.0 (0.3–1.8)	.30
ROSC to MRI	2.8 (2.0–4.0)	2.6 (1.9–3.8)	2.9 (2.0–4.1)	.42	2.0 (1.6–3.3)	2.8 (1.9–5.8)	.42
ROSC to obtain biomarker samples	4.6 (3.4–6.0), 84^c^	4.1 (3.2–5.8), 35^c^	4.7 (4.0–6.0), 49^c^	.13	3.3 (3.0–5.6), 47^c^	4.7 (3.2–5.9), 37^c^	.17
*Neuro-prognostication, median (IQR)*							
Presence of HSI on DW-MRI, *n* (%)	46 (41.8)	0	46 (74.2)	< 0.001			
Serum NSE, ng/mL	32.0 (21.8–57.6)	25.2 (19.2–32.2)	61.3 (42.1–152.8)	< 0.001	25.2 (19.2–32.2)	61.3 (42.1–152.8)	< 0.001
CSF NSE, ng/mL, 89^c^	44.4 (20.2–130.5)	23.5 (15.6–47.4)	130.0 (58.0–213.0)	< 0.001	23.5 (15.6–47.4)	130.0 (58.0–213.0)	< 0.001
Average ADC value, × 10^−6^ mm^2^/s	828.3 (774.6–853.4)	847.3 (829.4–867.4)	783.2 (647.5–829.2)	< 0.001	843.2 (828.4–865.4)	764.3 (593.1–806.7)	< 0.001
PV 650, %	17.1 (12.1–29.2)	13.2 (10.3–17.0)	31.1 (23.2–66.3)	< 0.001	13.2 (10.3–17.0)	31.1 (23.2–66.3)	< 0.001
GWR, 95^c^	1.22 (1.16–1.28)	1.23 (1.19–1.30)	1.20 (1.11–1.25)	< 0.001	1.24 (1.19–1.30)	1.20 (1.11–1.25)	.005
Low flow time, min	20.0 (10.0–30.0)	14.0 (8.0–20.8)	30.5 (22.8–44.5)	< 0.001	14.0 (8.0–20.8)	30.5 (22.8–44.5)	< 0.001
Q_A,_ 89^c^	0.008 (0.006–0.013)	0.007 (0.005–0.009)	0.011 (0.008–0.019)	< 0.001	0.007 (0.005–0.009)	0.011 (0.008–0.019)	< 0.001
*Outcome*				< 0.001			< 0.001
Survival	76 (69.7)	48 (100)	29 (46.8)		60 (95.2)	16 (34.8)	
Brain death	12 (11.0)	0	12 (19.4)		1 (1.6)	11 (23.9)	
WLST after 72 h from ROSC	6 (5.5)	0	6 (9.7)		0	6 (13.0)	

*IQR* interquartile range; *CCI* Charlson comorbidity index; *CPR* cardiopulmonary resuscitation; *PCI* percutaneous coronary intervention; *MRI* magnetic resonance imaging; *ROSC* return of spontaneous circulation; *CT* computed tomography; *HSI* high-signal intensity, *DW-MRI* diffusion-weighted magnetic resonance imaging; NSE neuron-specific enolase; *CSF* cerebrospinal fluid; *ADC* apparent diffusion coefficient; *PV 650* the percentage of voxels below 650 × 10^−6^ mm^2^/s in apparent diffusion coefficient; *GWR* gray-white matter ratio; *Q*_A_ albumin quotients; *WLST* withdrawal of life-sustaining treatment; *ROSC* return of spontaneous circulation

^a^P_HSI_, presence of HSI on DW-MRI; A_HSI_, absence of HSI on DW-MRI

^b^*P* values are based on *χ*^2^ test for categorical variables and Mann–Whitney U test for continuous variables

^c^Number of patients included in the analysis

### Analysis of ultra-early DW-MRI findings

P_HSI_ on DW-MRI was observed in 46 (41.8%) patients. Good neurological outcomes were observed in 0 (0%) patients in the P_HSI_ group and in 48 (75%) patients in the A_HSI_ group (Table 2, Fig. 3). Compared with the A_HSI_ group, the P_HSI_ group had lower rates of witnessed events, shockable rhythm, cardiac etiology, lower pH, longer low flow times (27.5 [20.0–39.8] min vs. 15.0 [9.0–18.8] min, respectively; *P* < 0.001), and lower average ADC value (764.3 [593.1–806.7] × 10^−6^ mm^2^/s vs. 843.2 [828.4–865.4] × 10^−6^ mm^2^/s, respectively; *P* < 0.001; Table 2). No differences were found between the groups in terms of age, sex, CCI, bystander CPR, and ROSC to MRI scan time (Table 2). In addition, using ROC analysis, the cut-off value of the average ADC value at 100% specificity for the presence of HSI in ultra-early DW-MRI is 760.5 × 10^–6^ mm/s (AUC, 0.89; 95% CI 0.79–0.93 and sensitivity, 47.8%; 95% CI 34.1%–61.9%) (see Additional file 1: Table S2).

### Association of single predictors with neurological outcome in A_HSI_ group

**Fig. 4:**
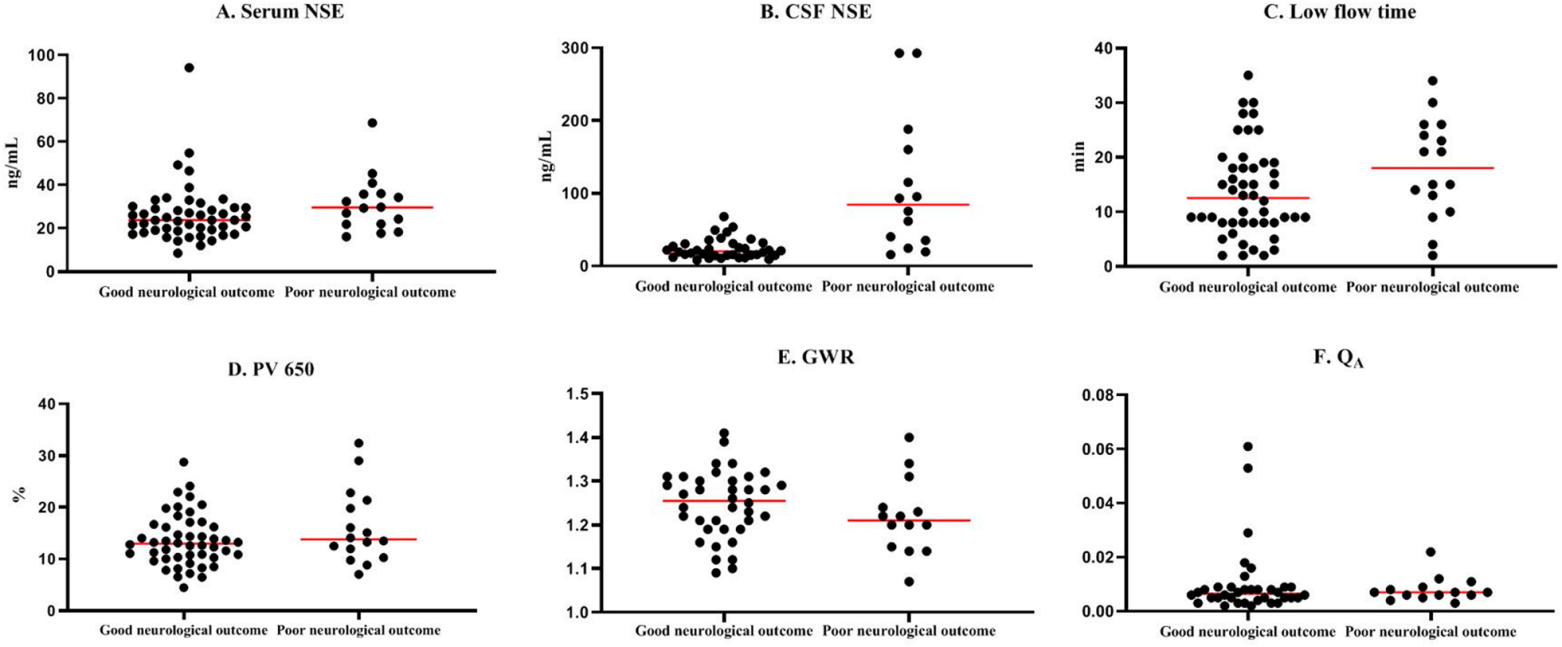
Association of single predictors with neurological outcome in A_HSI_ group. Red lines are the median value. *Abbreviations:* *A*_HSI_, absence of high-signal intensity; CSF, cerebrospinal fluid; *GWR,* gray-white matter ratio; *NSE*, neuron-specific enolase; *PV 650*, percentage of voxels below 650 × 10^−6^ mm^2^/s; *Q*_A_, albumin quotient (albumin_[CSF]_/albumin_[serum]_)

Upon analysis of the association of single predictors with neurological outcome in the A_HSI_ group, there were 48 (75%) and 16 (25%) patients with good and poor neurological outcomes, respectively (Fig. 3). Serum NSE levels (23.8 [18.9–29.5] ng/mL vs. 29.6 [21.9–36.0] ng/mL, *P* = 0.08), GWR (1.26 [1.19–1.30] vs. 1.21 [1.15–1.26], *P* = 0.23), PV 650 (13.2 [10.3–17.0] % vs. 13.2 [10.3–17.0] %, *P* = 0.28), low flow time (30.5 [22.8–44.5] min vs. 18.0 [10.8–25.5] min, *P* = 0.08), and *Q*_A_ (0.007 [0.005–0.009] vs. 0.007 [0.006–0.009], *P* = 0.61) showed no difference between the good and poor neurological outcome groups (Fig. 4). However, only CSF NSE levels were significantly lower in the good neurological outcome group compared with those of poor neurological outcome group (20.1 [14.4–30.7] ng/mL vs. 84.3 [32.4–167.0] ng/mL, *P* < 0.001; Fig. 4).

### Prognostic performance for neurological outcome using DW-MRI alone or combination

The AUC value of DW-MRI showed fair-to-excellent prognostic performance (AUC, 0.87; 95% CI, 0.79–0.92). Sensitivity, specificity, NPV, and PPV for predicting a poor neurological outcome were 74.2% (95% CI 62.0–83.5), 100% (95% CI 91.2–100.0), 75.0% (95% CI 63.1–84.1) and 100% (95% CI 90.8–100.0), respectively. On the other hand, the AUC value of the CSF NSE level showed good-to-excellent prognostic performance (AUC, 0.92; 95% CI, 0.84–0.97; Table 3), but at 100% (95% CI 88.5–100.0) specificity, the sensitivity was 67.9% (95% CI 54.5–79.0), which was lower than that of DW-MRI (Table 3).

**Table 3:** Prognostic performance of single predictors for poor neurological outcome

Predictor	Cut-off value	AUC (95% CI)	Sensitivity (95% CI)^c^	Specificity (95% CI)	PPV (95% CI)	NPV (95% CI)
DW-MRI	Presence of HSI	0.87 (0.79–0.93)	74.2 (62.0–83.5)	100.0 (91.2–100.0)	100.0 (90.8–100.0)	75.0 (63.1–84.1)
Serum NSE levels	> 94.2 ng/mL	0.81 (0.72–0.88)	22.6 (13.8–34.5)	100.0 (91.2–100.0)	100.0 (74.9–100.0)	50.0 (40.2–59.8)
CSF NSE levels, 89^a^	> 67.9 ng/mL	0.92 (0.84–0.97)	67.9 (54.5–79.0)	100.0 (88.5–100.0)	100.0 (88.5–100.0)	67.9 (54.5–79.0)
PV 650^b^	> 28.8%	0.83 (0.75–0.90)	48.4 (36.4–60.6)	100.0 (91.2–100.0)	100.0 (86.5–100.0)	60.0 (49.0–70.0)
GWR, 95^a^	≤ 1.07	0.68 (0.58–0.77)	14.0 (7.0–25.6)	100.0 (89.1–100.0)	100.0 (62.8–100.0)	43.7 (33.7–54.2)
Low flow time	> 35 min	0.82 (0.74–0.89)	30.6 (20.5–43.0)	100.0 (91.2–100.0)	100.0 (80.2–100.0)	52.7 (42.6–62.7)
Q_A_, 89 ^a^	> 0.061	0.70 (0.60–0.80)	3.8 (0.3–13.5)	100.0 (88.5–100.0)	100.0 (29.0–100.0)	41.4 (31.6–51.9)

*AUC* the area under the ROC curves; *CI* confidence interval; *NPV* negative predictive value; *PPV* positive predictive value; *DW-MRI* diffusion-weighted magnetic resonance imaging; *NSE* neuron-specific enolase; *CSF* cerebrospinal fluid; *GWR* gray-white matter ratio; *PV 650* the percentage of voxels below 650 × 10^−6^ mm^2^/s in apparent diffusion coefficient; *Q*_A_ albumin quotient

^a^Number of patients included in the analysis

^b^% Whole brain voxels with ADC below 650 × 10^−6^ mm^2^/s

In predicting poor neurological outcome prognosis in combination with DW-MRI, the combination of CSF NSE levels (AUC 0.97, 95% CI 0.90–0.99) had the highest prognostic performance, followed by serum NSE levels (AUC 0.91, 95% CI 0.84–0.96). At this time, when the false positive rate (FPR) was 0%, the sensitivity of CSF NSE levels and serum NSE levels were 88.7% (95% CI 77.1–95.1) and 72.6% (95% CI 60.3–82.2), respectively. The *Q*_A_ combination had the lowest prognostic performance (AUC 0.86, 95% CI 0.77–0.92) (Table 4).

**Table 4:** Prediction of poor neurological outcome at 6 months using DW-MRI and various predictor combinations

Combination	AUC (95% CI)	TP	TN	FP	FN	Sensitivity (95% CI)	Specificity (95% CI)	PPV (95% CI)	NPV (95% CI)
DW-MRI + Serum NSE	0.91 (0.84–0.96)	45	48	0	17	72.6 (60.3–82.2)	100.0 (91.2–100.0)	100.0 (90.6–100.0)	73.8 (62.0–83.1)
DW-MRI + CSF NSE, 89^a^	0.97 (0.90–0.99)	47	36	0	6	88.7 (77.1–95.1)	100.0 (88.5–100.0)	100.0 (91.0–100.0)	85.7 (71.8–93.7)
DW-MRI + PV 650	0.89 (0.82–0.95)	48	48	0	14	77.4 (65.5–86.2)	100.0 (91.2–100.0)	100.0 (91.2–100.0)	77.4 (65.5–86.2)
DW-MRI + GWR, 95^a^	0.90 (0.83–0.96)	44	38	0	13	77.2 (64.7–86.3)	100.0 (89.1–100.0)	100.0 (90.4–100.0)	74.5 (61.0–84.6)
DW-MRI + Low flow time	0.91 (0.84–0.96)	46	48	0	16	74.2 (62.0–83.5)	100.0 (91.2–100.0)	100.0 (90.8–100.0)	75.0 (63.1–84.1)
DW-MRI + Q_A_, 89^a^	0.86 (0.77–0.92)	39	36	0	14	73.6 (60.3–83.7)	100.0 (88.5–100.0)	100.0 (89.3–100.0)	72.0 (58.2–82.6)

*AUC* area under curve; *TP* true positive; *TN* true negative; *FP* false negative; *FN* false positive; *CI* confidence interval; *NPV* negative predictive value; *PPV* positive predictive value; *DW-MRI* diffusion-weighted magnetic resonance imaging; *NSE* neuron-specific enolase; *CSF* cerebrospinal fluid; *PV 650* the percentage of voxels below 650 × 10^−6^ mm^2^/s; *GWR* gray-white matter ratio; *Q*_A_ albumin quotient

^a^Number of patients included in the analysis

## Discussion

This study focused on the presence or absence of HSI in ultra-early DW-MRI performed in comatose OHCA survivors. All P_HSI_ group patients showed poor neurological outcomes, whereas 75% of the A_HSI_ group patients showed good neurological outcomes. A poor neurological outcome could be predicted without FPR predictions in the P_HSI_ group using ultra-early DW-MRI, with high sensitivity (74.2%, 95% CI 62.0–83.5). In addition, a higher predictive performance (AUC, 0.97; 95% CI 0.90–0.99) and sensitivity (88.7%, 95% CI 77.1%–95.1%) without FPR was observed in this group when combined with CSF NSE levels.

During the recent COVID-19 pandemic, patients with good neurological outcomes may have been deprived of treatment opportunities due to ICU overcrowding [30]; therefore, it is increasingly necessary to predict neurological outcomes prior to TTM for an appropriate distribution of medical resources. Several studies have been conducted to predict neurological outcomes early in comatose CA survivors, mostly using clinical variables and electroencephalography (EEG) findings [10–14]. However, there are some limitations in applying the results of these studies to clinical practice. First, it is challenging to accurately measure clinical data such as the time from CA to the start of basic life support (BLS), and BLS quality was not considered [31]. Second, most studies using EEG made assessments at least 12–24 h after ROSC, and their interpretations were complex and prone to subjectivity [14, 32]. Therefore, EEG is not suitable for determining medical resource distribution, such as the use of ICUs before TTM. Third, it is important to achieve a FPR of zero because poor outcome predictors can be used to determine the WLST; but high sensitivity at FPR 0% is required to be useful as a predictive tool [33]. However, in the recently reported study, the external validation of the 2020 ERC/ESICM prognostic strategy algorithm after cardiac arrest, a FPR of 0% was achieved; but the sensitivity was at the level of 60% [34]. In this study, DW-MRI provided results within 6 h after ROSC, did not require specific expertise to discriminate P_HSI_ only, and showed a sensitivity of 74.2% at FPR 0% to predict poor neurological outcome. In addition, when combined with CSF NSE levels, the sensitivity rises to 88.7%.

In patients with cardiac arrest, ischemia at the cellular level results in cessation of aerobic metabolism with consequent depletion of the high-energy substrate adenosine triphosphate (ATP) [35]. At this time, ATP depletion causes dysfunction of the energy-dependent Na^+^/K^+^ ion exchange pump action, resulting in massive sodium and water influx and intracellular cytotoxic edema [32]. According to previously published animal experiments, cerebral edema occurred during cardiac arrest and resuscitation, and the average ADC value decreased by more than 60% from normal, but it was reported that it returned to normal 30 min after ROSC [36]. However, in the group with low initial reperfusion pressure or non-sustained survival, the average ADC value did not recover to normal, and it was reported that ATP and glucose were depleted and lactate was severely increased compared to the group that recovered from bioluminescence imaging [36, 37]. It is presumed that the group with poor prognosis further exacerbated intracellular damage during ischemic and reperfusion injury and induced a vicious cycle leading to cell damage and death by causing energy failure [31, 36, 37]. In this study, the average ADC value of the P_HSI_ group was significantly lower than that of the A_HSI_ group (764.3 vs. 843.2 × 10^−6^ mm^2^/s, *P* < 0.001), and all of the P_HSI_ groups showed poor neurological outcomes. Irreversible HIBI can be assumed to have occurred if HSI is present on the ultra-early DW-MRI, regardless of location and amount. In addition, the cut-off value of the average ADC value at 0% FPR (sensitivity 47.8%) for the P_HSI_ in ultra-early DW-MRI was 760.5 × 10^–6^ mm/s.

Cytotoxic edema due to brain injury after acute CA shows HSI in DW-MRI with corresponding low ADC from a very early time [32, 33, 38, 39]. On the other hand, it is a potentially valuable predictor of good neurological outcome if A_HSI_ is observed on DW-MRI. However, compared to other studies, only our previous and this study have shown 100% specificity when predicting poor neurological outcomes using the presence or absence of HSI in DW-MRI [17, 18]. We speculated different results based on determining the single or multi-focal HSI results in DW-MRI as positive/negative. The reason we excluded these findings from this study can be explained as follows. First, Oh et al. have reported that single focal HSI or absence of HSI in DW-MRI performed immediately after the rewarming phase of TTM showed a good neurological outcome [23]. Second, our previous studies showed that, if HSI was present in ultra-early DW-MRI, the HSI area appeared to expand in DW-MRI after 72 h of ROSC, which was because the occurrence of brain edema post-CA brain injury was time-dependent [9, 18]. That is, there is a difference between single lesion or multiple HSI which corresponds to specific vascular territories as opposed to a diffuse spread throughout the cerebral cortex or deep gray matter that can be viewed as post-CA brain injury. In the present study, four patients with 1–2 focal HSI observed in ultra-early DW-MRI were excluded as undetermined. All of them showed good neurological outcomes with 1–2 focal HSIs in which the HSI area had not expanded, on DW-MRI 3–4 days after ROSC. However, our conclusion that focal HSI shows good neurological outcomes cannot be generalized as our study involved only a small number of patients from a single center. Nevertheless, if neurological outcomes following DW-MRI are to be predicted according to the presence or absence of HSI, we consider it advisable to exclude focal HSIs as being undetermined until the results of a multicenter large-scale study are obtained and to use other predictive tools in the meantime.

International guidelines for post-CA care recommend a multi-modal approach to predict prognosis [5, 6]. However, a combination of various predictors does not unconditionally increase the predictive performance, sensitivity, or specificity [34]. In our study, the DW-MRI and CSF NSE levels combination had better predictive performance than DW-MRI alone or other combination. Thus, we consider only the CSF NSE level to be significantly different from the neurological outcome in the A_HSI_ group compared to other predictors. We speculate that this outcome is related to the degree of BBB disruption [19, 24]. The P_HSI_ group showed moderate BBB disruption (median value Q_A_, 0.011), and the A_HSI_ groups showed no BBB disruption (an upper normal margin, and a median Q_A_ of 0.007). In our previous studies, we reported a difference in CSF NSE levels between good and poor neurological outcomes when BBB disruption did not occur, but no differences in serum NSE levels [19]. In addition, Geocardin et al. reported that CSF samples have the advantage of the biomarker not requiring to be transported across the BBB for detection, thereby greatly reducing the contamination issue [40].

In this study, we focused on predicting poor neurological outcome at 6 months, following the structuring of most of the literature. However, we suggest that early (i.e., before TTM) prediction of outcome in cardiac arrest survivors focus on good rather than poor neurological outcomes. Recently, Sandroni et al. in their study "Accuracy for prediction of good outcome corresponds to the inverse of their accuracy for prediction of poor outcome" reported that the specificity for prediction of good neurological outcome corresponds to the sensitivity for prediction of poor neurological outcome, and vice versa [9]. Therefore, it is assumed that DW-MRI alone or the combination of DW-MRI and CSF NSE can predict good neurological outcome six months after cardiac arrest with high specificity without false negative rate.

## Limitations

Our study had several important limitations. First, this retrospective, single center study had a small number of patients and the selected threshold or other statistical outcomes in this study may have been affected; therefore, a multicenter study is needed to generalize the results. Second, self-fulfilling prophecy bias was possible as the treating physicians were exposed to the results of DW-MRI and CSF NSE levels. However, WLST was not permitted in South Korea prior to February 2018 unless a patient was diagnosed with brain death and, in this study, no patients underwent WLST during TTM. Third, this study measured CSF NSE levels and Q_A_ and evaluated its combination with DW-MRI findings. However, in CA survivors, lumbar puncture is invasive and rare in clinical practice, and MRI is known to be challenging when evaluating patients with unstable vital signs; hence, these procedures are generally not applied. However, in our previous study [41], the median ICP measured 4.5 h after ROSC in the good and poor neurological outcome groups was within a normal range (10.4 mmHg and 12.5 mmHg, respectively). In addition, MRI can be safely performed within a short time through applying a portable ventilator and patient monitoring, and through scanning only DW-MRI and ADC sequences. There were no patient safety concerns during MRI scans in this study. Fourth, a logistic regression analysis with cardiac arrest characteristics is essential to investigate independent effect of prognostic tests for neurological outcome. Unfortunately, odds that a good neurological outcome was exposed to HSI on DWI was zero. Thus, odds ratio of HSI on DW-MRI for outcome could not be calculated using logistic regression analysis, because all patients who showed HSI on DWI were determined as a poor neurological outcome. Finally, the cut-off value for combination models was not suggested in this study, because all the combination models were constructed using a predictive probability in the logistic regression analyses.

## Conclusion

P_HSI_ in ultra-early DW-MRI of OHCA survivors was significantly associated with a poor neurological outcome. In addition, the combination of CSF NSE levels showed higher sensitivity at 100% specificity than DW-MRI alone or other combinations. Further studies are needed to validate our findings.

## Supplementary Information

**Additional file 1**. 1. Definition of high-signal intensity in diffusion-weighted magnetic resonance imaging. 2. **Table S1:** Inter-rater reliability analysis of interpretations for DW-MRI between two experts. 3. **Figure S1**: Classification of hypoxic ischemic brain injury according to the lesion visualized on DW-MRI and corresponding ADC map. 4. **Table S2:** Prognostic performance of average ADC value for presence of high-signal intensity in ultra-early DW-MRI.

## Data Availability

The datasets used and/or analyzed during the current study are available from the corresponding author on reasonable request.

## Abbreviations

ADC: Apparent diffusion coefficient
A_HSI_: Absence of HSI
ATP: Adenosine triphosphate
AUROC: Area under the receiver operating characteristic curve
BBB: Blood–brain barrier
BLS: Basic life support
CA: Cardiac arrest
CC: Corpus callosum
CCI: Charlson comorbidity index score
CI: Confidence interval
CN: Caudate nucleus
CPC: Cerebral performance category
CPR: Cardiopulmonary resuscitation
CSF: Cerebrospinal fluid
CT: Computed tomography
DW-MRI: Diffusion-weighted magnetic resonance image
EEG: Electroencephalography
FPR: False positive rate
GWR: Gray-white matter ratio
HIBI: Hypoxic ischemic brain injury
HSI: High-signal intensity
HU: Hounsfield units
ICP: Intracranial pressure
ICU: Intensive care unit
IQR: Interquartile range
LP: Lumbar puncture
MRI: Magnetic resonance imaging
NPV: Negative predictive value
NSE: Neuron-specific enolase
OHCA: Out-of-hospital cardiac arrest
P: Putamen
P_HSI_: Presence of HSI
PIC: Posterior limb of the internal capsule
PPV: Positive predictive value
PV: Percentage of voxel
Q_A_: Albumin quotient (albumin_[CSF]_/albumin_[serum]_)
ROSC: Return of spontaneous circulation
TTM: Target temperature management
WLST: Withdrawal of life-sustaining treatment

## References

[1] Tsao CW, Aday AW, Almarzooq ZI, Alonso A, Beaton AZ, Bittencourt MS (2022). Heart disease and stroke statistics-2022 update: a report from the American Heart Association. Circulation.

[2] Callaway CW, Schmicker RH, Brown SP, Albrich JM, Andrusiek DL, Aufderheide TP (2014). Early coronary angiography and induced hypothermia are associated with survival and functional recovery after out-of-hospital cardiac arrest. Resuscitation.

[3] Elmer J, Torres C, Aufderheide TP, Austin MA, Callaway CW, Golan E (2016). Resuscitation outcomes consortium. Association of early withdrawal of life-sustaining therapy for perceived neurological prognosis with mortality after cardiac arrest. Resuscitation.

[4] Grossestreuer AV, Gaieski DF, Abella BS, Wiebe DJ, Moskowitz A, Ikeda DJ (2017). Factors associated with post-arrest withdrawal of life-sustaining therapy. Resuscitation.

[5] Nolan JP, Sandroni C, Böttiger BW (2021). European Resuscitation Council and European Society of Intensive Care Medicine guidelines 2021: post-resuscitation care. Intensive Care Med.

[6] Kim YM, Jeung KW, Kim WY, Park YS, Oh JS, You YH (2020). Korean guidelines for cardiopulmonary resuscitation. Part 5. Post-cardiac arrest care. Clin Exp Emerg Med..

[7] Son SH, Lee IH, Park JS, Yoo IS, Kim SW, Lee JW (2020). Does combining biomarkers and brain images provide improved prognostic predictive performance for out-of-hospital cardiac arrest survivors before target temperature management?. J Clin Med.

[8] Lee BK, Min JH, Park JS, Kang C, Lee BK (2022). Early identified risk factors and their predictive performance of brain death in out-of-hospital cardiac arrest survivors. Am J Emerg Med.

[9] Sandroni C, D'Arrigo S, Cacciola S, Hoedemaekers CWE, Westhall E, Kamps MJA (2022). Prediction of good neurological outcome in comatose survivors of cardiac arrest: A systematic review. Intensive Care Med.

[10] Hirano Y, Kondo Y, Sueyoshi K, Okamoto K, Tanaka H (2021). Early outcome prediction for out-of-hospital cardiac arrest with initial shockable rhythm using machine learning models. Resuscitation.

[11] Fung FW, Topjian AA, Xiao R, Abend NS (2019). Early EEG features for outcome prediction after cardiac arrest in children. J Clin Neurophysiol.

[12] Chen S, Lachance BB, Gao L, Jia X (2021). Targeted temperature management and early neuro-prognostication after cardiac arrest. J Cereb Blood Flow Metab.

[13] Eertmans W, Tran TMP, Genbrugge C, Peene L, Mesotten D, Dens J (2018). A prediction model for good neurological outcome in successfully resuscitated out-of-hospital cardiac arrest patients. Scand J Trauma Resusc Emerg Med.

[14] Oh SH, Park KN, Shon YM, Kim YM, Kim HJ, Youn CS (2015). Continuous amplitude-integrated electroencephalographic monitoring is a useful prognostic tool for hypothermia-treated cardiac arrest patients. Circulation.

[15] Hong JY, Lee DH, Oh JH, Lee SH, Choi YH, Kim SH (2019). Grey-white matter ratio measured using early unenhanced brain computed tomography shows no correlation with neurological outcomes in patients undergoing targeted temperature management after cardiac arrest. Resuscitation.

[16] In YN, Lee IH, Park JS, Kim DM, You Y, Min JH (2022). Delayed head CT in out-of-hospital cardiac arrest survivors: Does this improve predictive performance of neurological outcome?. Resuscitation.

[17] Jeon CH, Park JS, Lee JH, Kim H, Kim SC, Park KH (2017). Comparison of brain computed tomography and diffusion-weighted magnetic resonance imaging to predict early neurologic outcome before target temperature management comatose cardiac arrest survivors. Resuscitation.

[18] Park JS, In YN, You YH, Min JH, Ahn HJ, Yoo IS (2020). Ultra-early neurologic outcome prediction of out-of-hospital cardiac arrest survivors using combined diffusion-weighted imaging findings and quantitative analysis of apparent diffusion coefficient. Resuscitation.

[19] You Y, Park JS, Min J, Yoo I, Ahn HJ, Cho Y (2019). The usefulness of neuron-specific enolase in cerebrospinal fluid to predict neurological prognosis in cardiac arrest survivors who underwent target temperature management: A prospective observational study. Resuscitation.

[20] Muttikkal TJ, Wintermark M (2013). MRI patterns of global hypoxic-ischemic injury in adults. J Neuroradiol.

[21] Pai V, Sitoh YY, Purohit B (2020). Gyriform restricted diffusion in adults: Looking beyond thrombo-occlusions. Insights Imaging.

[22] Oren NC, Chang E, Yang CW, Lee SK (2019). Brain diffusion imaging findings may predict clinical outcome after cardiac arrest. J Neuroimaging.

[23] Oh SH, Park KN, Choi SP, Oh JS, Kim HJ, Youn CS (2019). Beyond dichotomy: Patterns and amplitudes of SSEPs and neurological outcomes after cardiac arrest. Crit Care.

[24] Park JS, You Y, Min JH, Yoo I, Jeong W, Cho Y (2019). Study on the timing of severe blood-brain barrier disruption using cerebrospinal fluid-serum albumin quotient in post cardiac arrest patients treated with targeted temperature management. Resuscitation.

[25] Rittenberger JC, Raina K, Holm MB, Kim YJ, Callaway CW (2011). Association between cerebral performance category, modified rankin scale, and discharge disposition after cardiac arrest. Resuscitation.

[26] DeLong ER, DeLong DM, Clarke-Pearson DL (1988). Comparing the areas under two or more correlated receiver operating characteristic curves: A nonparametric approach. Biometrics.

[27] Agresti A, Coull BA (1998). Approximate is better than “Exact” for interval estimation of binomial proportions. Am Stat.

[28] Muller MP, Tomlinson G, Marrie TJ, Tang P, McGeer A, Low DE, Detsky AS (2005). Can routine laboratory tests discriminate between severe acute respiratory syndrome and other causes of community-acquired pneumonia?. Clin Infect Dis.

[29] Landis JR, Koch GG (1977). The measurement of observer agreement for categorical data. Biometrics.

[30] Kim K, Ghorbanzadeh M, Horner MW, Ozguven EE (2021). Identifying areas of potential critical healthcare shortages: A case study of spatial accessibility to ICU beds during the COVID-19 pandemic in Florida. Transp Policy (Oxf).

[31] Tanguay-Rioux X, Grunau B, Neumar R, Tallon J, Boone R, Christenson J (2018). Is initial rhythm in OHCA a predictor of preceding no flow time? Implications for bystander response and ECPR candidacy evaluation. Resuscitation.

[32] Sandroni C, Cronberg T, Sekhon M (2021). Brain injury after cardiac arrest: pathophysiology, treatment, and Prognosis. Intensive Care Med.

[33] Sandroni C, D'Arrigo S, Cacciola S, Hoedemaekers CWE, Kamps MJA, Oddo M (2020). Prediction of poor neurological outcome in comatose survivors of cardiac arrest: A systematic review. Intensive Care Med.

[34] Youn CS, Park KN, Kim SH, Lee BK, Cronberg T, Oh SH (2022). External validation of the 2020 ERC/ESICM prognostication strategy algorithm after cardiac arrest. Crit Care.

[35] Sekhon MS, Ainslie PN, Griesdale DE (2017). Clinical pathophysiology of hypoxic ischemic brain injury after cardiac arrest: A "two-hit" model. Crit Care.

[36] Fischer M, Bockhorst K, Hoehn-Berlage M, Schmitz B, Hossmann KA (1995). Imaging of the apparent diffusion coefficient for the evaluation of cerebral metabolic recovery after cardiac arrest. Magn Reson Imaging.

[37] Hossmann KA, Fischer M, Bockhorst K, Hoehn-Berlage M (1994). NMR imaging of the apparent diffusion coefficient (ADC) for the evaluation of metabolic suppression and recovery after prolonged cerebral ischemia. J Cereb Blood Flow Metab.

[38] Hirsch KG, Fischbein N, Mlynash M, Kemp S, Bammer R, Eyngorn I (2020). Prognostic value of diffusion-weighted MRI for post-cardiac arrest coma. Neurology.

[39] Michinaga S, Koyama Y (2015). Pathogenesis of brain edema and investigation into anti-edema drugs. Int J Mol Sci.

[40] Geocadin RG, Callaway CW, Fink EL, Golan E, Greer DM, Ko NU (2021). Standards for studies of neurological prognostication in comatose survivors of cardiac arrest: A scientific statement from the American Heart Association. Circulation.

[41] Song H, Kang C, Park J, You Y, In Y, Min J (2021). Intracranial pressure patterns and neurological outcomes in out-of-hospital cardiac arrest survivors after targeted temperature management: A retrospective observational study. J Clin Med.

