# Supplementary material for: Association of ultra-early diffusion-weighted magnetic resonance imaging with neurological outcomes after out-of-hospital cardiac arrest
Source: Crit Care. 2023 Jan 13;27:16. doi: 10.1186/s13054-023-04305-z (PMC9837995; doi:10.1186/s13054-023-04305-z)
Supplement: Supplementary file 1 — Additional file 1. 1. Definition of high-signal intensity in diffusion-weighted magnetic resonance imaging. 2. Table S1: Inter-rater reliability analysis of interpretations for DW-MRI between two experts. 3. Figure S1: Classification of hypoxic ischemic brain injury according to the lesion visualized on DW-MRI and corresponding ADC map. 4. Table S2: Prognostic performance of average ADC value for presence of high-signal intensity in ultra-early DW-MRI. [file 13054_2023_4305_MOESM1_ESM.docx]

**Supplementary materials**

**Title: Association of ultra-early diffusion-weighted magnetic resonance imaging with neurological outcome after out-of-hospital cardiac arrest**

**1. Definition of high signal intensity in diffusion-weighted magnetic resonance imaging**

P_HSI_ was defined as a diffuse HSI on DW-MRI according to previous studies for MRI patterns of global hypoxic-ischemic injury. P_HSI_ was a restricted diffusion on DW-MRI with corresponding hypoattenuation on the ADC map irrespective of volume. In detail, gyriform restricted diffusion in the cerebral cortex on DW-MRI was considered as P_HSI_, and HSI in deep gray matter on DW-MRI was also considered P_HSI_ because deep gray matter is known as vulnerable area to hypoxic-ischemic injury.^1, 2^ The pattern of HSI in DW-MRI which is not suggesting HIBI due to cardiac arrest (e.g., non-gyriform restricted diffusion as a single lesion or multiple foci, which corresponds to specific vascular territories) were excluded in this analysis.

**2. Table S1. Inter-rater reliability analysis of interpretations for DW-MRI between two experts**

| **Modality** | **Reviewer 1** | **Reviewer 2** | **Kappa value** |
| --- | --- | --- | --- |
| DW-MRI |  |  |  |
| P_HSI_, n (%) | 46 (41.8) | 53 (48.2) | 0.87 |

**Abbreviations:** DW-MRI, diffusion weighted magnetic resonance imaging; P_HSI_, presence of high signal intensity

**3. Figure S1. Classification of hypoxic ischemic brain injury according to the lesion visualized on DW-MRI and corresponding ADC map**


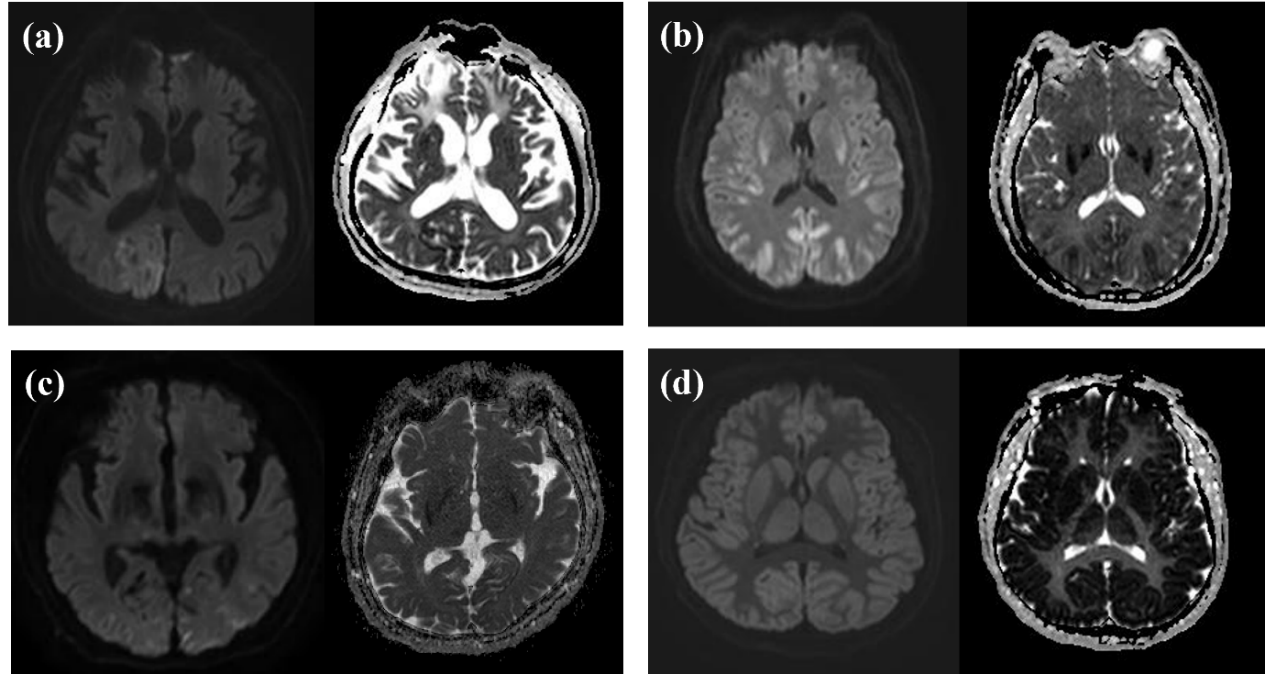


Group (a): Regional involvement: RD in cortical or deep grey matter involving a particular region of a unilateral hemisphere

Group (b): Multi-regional involvement: RD in cortical or deep grey matter involving 2 or more regions of cerebral hemispheres

Group (c): Multi-focal pattern: Scattered, discrete foci of RD

Group (d): Global involvement: RD with global cortical and deep grey matter involvement

DW-MRI, diffusion weighted magnetic resonance imaging; ADC, apparent diffusion coefficient; RD: Restricted diffusion

**4. Table S2. Prognostic performance of average ADC value for presence of high-signal intensity in ultra-early DW-MRI**

| Characteristics | Cut-off value | AUC (95% CI) | Specificity (95% CI) | Sensitivity (95% CI) | NPV (95% CI ) | PPV (95% CI) |
| --- | --- | --- | --- | --- | --- | --- |
| Average ADC value | ≤ 760.5 × 10^−6^ mm^2^/s | 0.89 (0.79 – 0.93) | 100.0 (94.4 – 100.0) | 47.8 (34.1 – 61.9) | 72.7 (62.6 – 81.0) | 100.0 (82.5 – 100.0) |

**Abbreviations**: AUC, the area under the ROC curves; CI, confidence interval; NPV, negative predictive value; PPV, positive predictive value; DW-MRI, diffusion weighted magnetic resonance imaging; ADC, apparent diffusion coefficient

Reference

1. Oren NC, Chang E, Yang CW, Lee SK. Brain Diffusion Imaging Findings May Predict Clinical Outcome after Cardiac Arrest. *J Neuroimaging*. 2019;29(4):540-547.
2. Pai V, Sitoh YY, Purohit B. Gyriform restricted diffusion in adults: looking beyond thrombo-occlusions. Insights Imaging. 2020;11(1):20.
